# Zinc Oxide Films Fabricated via Sol-Gel Method and Dip-Coating Technique–Effect of Sol Aging on Optical Properties, Morphology and Photocatalytic Activity

**DOI:** 10.3390/ma16051898

**Published:** 2023-02-24

**Authors:** Katarzyna Wojtasik, Magdalena Zięba, Cuma Tyszkiewicz, Wojciech Pakieła, Grażyna Żak, Olgierd Jeremiasz, Ewa Gondek, Kazimierz Drabczyk, Paweł Karasiński

**Affiliations:** 1 Department of Optoelectronics, Silesian University of Technology, B. Krzywoustego 2, 44-100 Gliwice, Poland; 2 Department of Engineering Materials and Biomaterials, Silesian University of Technology, Konarskiego 18a, 44-100 Gliwice, Poland; 3 Oil and Gas Institute–National Research Institute, Lubicz 25A, 31-503 Kraków, Poland; 4 Institute of Metallurgy and Materials Science, Polish Academy of Sciences, Reymonta 25, 30-059 Kraków, Poland; 5 Helioenergia Sp. z o.o., Rybnicka 68, 44-238 Czerwionka-Leszczyny, Poland; 6 Department of Physics, Cracow University of Technology, Podchorążych 1, 31-084 Kraków, Poland

**Keywords:** sol-gel, dip-coating, zinc oxide, thin layers, energy band gap, hydrophilicity, photocatalytic activity

## Abstract

Zinc oxide layers on soda-lime glass substrates were fabricated using the sol-gel method and the dip-coating technique. Zinc acetate dihydrate was applied as the precursor, while diethanolamine as the stabilizing agent. This study aimed to determine what effect has the duration of the sol aging process on the properties of fabricated ZnO films. Investigations were carried out with the sol that was aged during the period from 2 to 64 days. The sol was studied using the dynamic light scattering method to determine its distribution of molecule size. The properties of ZnO layers were studied using the following methods: scanning electron microscopy, atomic force microscopy, transmission and reflection spectroscopy in the UV-Vis range, and the goniometric method for determination of the water contact angle. Furthermore, photocatalytic properties of ZnO layers were studied by the observation and quantification of the methylene blue dye degradation in an aqueous solution under UV illumination. Our studies showed that ZnO layers have grain structure, and their physical–chemical properties depend on the duration of aging. The strongest photocatalytic activity was observed for layers produced from the sol that was aged over 30 days. These layers have also the greatest porosity (37.1%) and the largest water contact angle (68.53°). Our studies have also shown that there are two absorption bands in studied ZnO layers, and values of optical energy band gaps determined from positions of maxima in reflectance characteristics are equal to those determined using the Tauc method. Optical energy band gaps of the ZnO layer fabricated from the sol aged over 30 days are *E_g_^I^* = 4.485 eV and *E_g_^II^* = 3.300 eV for the first and second bands, respectively. This layer also showed the highest photocatalytic activity, causing the pollution to degrade 79.5% after 120 min of UV irradiation. We believe that ZnO layers presented here, thanks to their attractive photocatalytic properties, may find application in environmental protection for the degradation of organic pollutants.

## 1. Introduction

Zinc oxide (ZnO) has attractive physicochemical properties for many applications in modern technology, which determines its high application potential. It is a wide-band gap oxide semiconductor with a direct energy gap. The wide energy gap of bulk zinc oxide (*Eg_dir_* = 3.37 eV) causes it to have good transmission properties in the visible range of electromagnetic radiation. It has high exciton binding energy (~60 meV) at room temperature [1]. It is a non-toxic, chemically stable material with sensor properties and high photocatalytic activity [2]. Depending on manufacturing technology, fabricated ZnO samples can have different morphology. Zero-dimensional, 1D, 2D, and 3D dimensional structures can be produced [3,4,5,6]. ZnO films are currently being intensively developed for applications in optoelectronics [7], in chemical sensors as sensor layers [8], and for use as protective coatings [9]. Considering optoelectronics, photovoltaics is currently the primary area of ZnO application. ZnO layers are used as photoanodes of DSSCs (Dye-Sensitized Solar Cells) [10]. Moreover, ZnO layers in solar cells, thanks to their good transmission properties and appropriate electrical properties, are used as TCO (Transparent Conductive Oxide) layers [11]. They are successfully replacing the previously used ITO layers. Finally, zinc oxide layers are used as ETL (Electron Transport Layer) in perovskite solar cells [12]. This article focuses on the fabrication and characterization of zinc oxide thin layers, with particular emphasis put on investigations of the effect sol aging has on the morphology, optical, and photocatalytic properties of ZnO layers.

Many methods are used to produce ZnO thin layers, including atomic layer deposition (ALD) [13], sputtering [14], spray pyrolysis [8], electrodeposition [15], microwave assisted synthesis [16], and sol-gel [17]. Innovative methods are also used, e.g., simultaneous etching and Al doping of H_2_O-oxidized ZnO nanorods to generate Al-doped ZnO nanotubes [18]. The applied method affects the morphology and properties of the ZnO layers produced [19,20]. The sol-gel method offers the greatest opportunities in terms of shaping the structure of the produced material. From a technical point of view, the sol-gel method may seem like a simple method, however, the development of layers with the desired properties requires an understanding of the phenomena occurring in the processes of sol synthesis and layering [21]. It is necessary to carry out many time-consuming studies on the influence of the composition of the sols, the parameters of the hydrolysis and condensation processes, the conditions of layer deposition (e.g., the withdrawal speed, which is the fundamental parameter of the dip-coating method), and the conditions of their annealing on the final properties of the layers. The subjects of the research are the type of precursor and its concentration, chelating and stabilizing agents, the temperature of sol synthesis, annealing of sol layers, the nature of the solvent, and the conditions of sol aging [22,23,24,25]. All these factors affect the structure, properties, and morphology of materials produced by the sol-gel method. This paper presents the results of our research on the production of ZnO layers by the sol-gel method and the dip-coating technique, with particular emphasis on the influence of the sol aging time on their properties.

Numerous research groups have reported results of studies on the influence of the duration of sol aging processes on properties of ZnO layers fabricated from sols [17,23,26,27,28,29,30]. As one can notice, studies were carried out for sols aged from several days [17] to several dozen days [23]. Aghkonbad et al. [29] studied the influence of the duration of sol aging processes, lasting in the range of 0–36 h, on the properties of layers produced from aged sols. They reported that ZnO layers, deposited on glass substrates, fabricated from the sol aged in this range of time, have high quality and were characterized by the highest transmission in the UV-Vis range, while their optical bandgap was broadened. Marouf et al. in Ref. [27] reported the synthesis of a sol without the addition of a stabilizing agent. They examined the sol during the period of 13 days. They found that the optimal aging time is 7 days, after which the sol reached stability, and the layers from that day were characterized by high transmittance (exceeding 70% in the visible range) and smooth surface. Toubane et al. reported in Ref. [23] studies in which sols were aged over the longest period, 30 days. They concluded that, as a result of increasing the duration of the sol aging process, fabricated ZnO layers have better transmittance in the UV-Vis range (up to 96%), increased optical band gap *Eg*, and improved smoothness. That group also reported investigations of the influence of the duration of sol aging on the photocatalytic activity, as well as the results of their visual assessment during their aging. Considering sols, they were synthesized from zinc acetate dihydrate (precursor), both without a stabilizing agent [27] and with the addition of DEA [23].

Zinc oxide has very good photocatalytic properties. It is frequently used by researchers for the photodegradation of pollutants because it has very good stability and availability, and the mobility of electrons in ZnO is high [19]. The latest literature reports indicate a wide interest in modified ZnO materials due to the possibility of shifting the spectral range of absorbed electromagnetic radiation in the presence of a certain photocatalyst. This is achieved by doping ZnO with metals or non-metals [31], or by producing hybrid structures with other metal oxides [32] or nanocomposites [33,34,35]. Excellent photocatalytic and antibacterial activity was also observed for hybrid systems of zinc oxide nanoparticles with reduced graphene oxide and gold nanoparticles [36]. However, the knowledge of the dependence of photocatalytic activity on the structure of pure ZnO is crucial to conduct more complex experiments.

This work presents the results of research on the influence of the duration of the sol aging process on the properties of ZnO layers. The sol was aged for 64 days, and ZnO layers were fabricated during this period. The sol was synthesized using zinc acetate dihydrate as the precursor, ethanol as the solvent, and DEA as a stabilizing agent. Glass substrates were coated with the single sol layer using the dip-coating technique. The subjects of research presented in this paper are the temporal stability of the sol, the optical properties of pure ZnO layers, produced from that sol, as well as their morphology and photocatalytic activity. The latter was determined from observations of the degradation of methylene blue (MB) using the Langmuir–Hinshelwood method. The influence of sol aging time on transmittance, optical band gap, Urbach energy, porosity, morphology, and surface roughness of produced ZnO layers was determined. The diameters of the nanocrystals were estimated from the transmission spectra in the absorption region by the analysis of the quantum size effect. The hydrophilic properties of the produced ZnO films were also studied. So far, investigations of the aforementioned properties of ZnO layers carried out for so lengthy duration of the sol aging process have not been published. We found that parameters of the ZnO layer fabricated from the sol aged for 30 days, take extreme values.

## 2. Materials and Methods

### 2.1. Materials

Zinc oxide layers were produced by the sol-gel method and the dip-coating technique. The sol was prepared by a procedure similar to that reported by Liu et al. in Ref. [37]. Zinc acetate dihydrate (Zn(CH_3_COO)_2_‧2H_2_O, ZAD, purchased from Sigma-Aldrich, Steinheim-Germany) as a precursor of zinc oxide, nonaqueous ethanol (EtOH, 99.8%, Avantor Performance Materials, Gliwice-Poland) as a solvent, and diethanolamine (DEA, Sigma-Aldrich, Steinheim-Germany) as a chelating agent. Deionized water was used directly from the deionizer (Polwater DL2-100S613TUV, Labopol Solution&Technologies, Kraków, Poland). All reagents were used without prior purification. The sol obtained in this way was filtered through a 0.2 μm PTFE syringe filter (Puradisc 25 TF, Whatman, Maidstone, UK).

### 2.2. Methods

If zinc acetate dihydrate (ZAD) is used as the precursor of ZnO, sols can be prepared in two ways. One can form them in (i) an acid medium in the system ethanol-acetic acid-water-ZAD [38], or (ii) a mild alkaline medium ethanol–DEA–ZAD [39]. The choice of the reaction method (method (i) or (ii)) affects the structure of the material produced, the surface morphology, and the surface properties of the produced layers [1]. In the studies reported here, we used method (ii) due to the slower gelation time, which probably results, among others, from the complexation of Zn(II) ions by DEA [23]. A strong, stable bond is formed between DEA and Zn^2+^, so the gelation process is slower.

**Figure 1:**
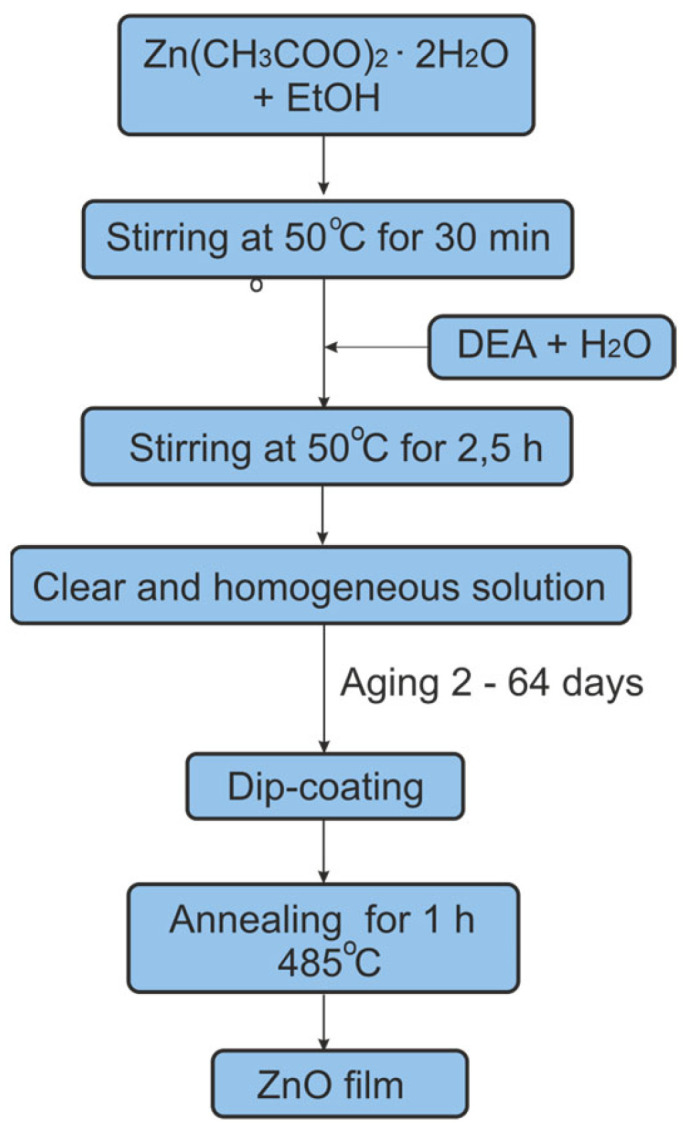
The course of the procedure for producing thin ZnO films.

The scheme of the ZnO layer fabrication process is shown in Figure 1. A portion of ZAD was added to 50 mL of ethanol and stirred at (50 °C) for 30 min, resulting in a 0.6 mol/L solution, to which DEA and H_2_O were added. The molar ratios of ZAD:DEA and ZAD:H_2_O were 1:0.6 and 1:2, respectively. The solution was then stirred in an ultrasonic bath for 2.5 h at 50 °C. A clear solution was obtained. It was subsequently filtered through a 0.2 μm PTFE syringe filter. The sol prepared in this way, closed in a vessel, was stored at a temperature of 18 °C. Soda-lime glass microscope slides (Menzel Gläser, Thermo Scientific), of dimensions 76 × 26 × 1 mm^3^, were coated with sol films after a certain elapsed time. Glass substrates were cleaned according to the procedure described in Ref. [30]. All ZnO layers were produced at the same withdrawal speed of the substrates from sol of 4.7 cm/min. Each substrate after deposition of the layer was annealed at 485 °C for 60 min.

ZnO powder was prepared for FTIR measurements. For this purpose, 3 mL of the sol was annealed at 485 °C for 60 min. The resulting powder had a gray color.

The size distribution of particles in sol was determined using the dynamic light scattering (DLS) method. The DLS was measured at a fixed angle of *θ* = 173° using a Zetasizer Nano (Malvern, Worcestershire, UK) with a laser beam of wavelength *λ_0_* = 633 nm. The IR spectra of ZnO powder were recorded in the frequency range of 400–4000 cm^−1^ using the Thermo Nicolet i-5 apparatus (Thermo Fisher Scientific, Waltham, MA, USA) with an ATR attachment. The thicknesses and refractive indices of ZnO layers were measured by monochromatic ellipsometry using the SE400 ellipsometer (Sentech, model 2003, *λ* = 632.8 nm, Berlin, Germany). Transmittance and reflectance spectra were recorded using the UV-Vis AvaSpec-ULS2048LTEC Spectrophotometer (Avantes, Apeldoorn, The Netherlands) and Lab-grade Reflection Probes QR400-7-SR (Ocean Optics, Orlando, FL, USA). The AvaLight-DH-S-BAL (Avantes, Apeldoorn, The Netherlands) was used as a light source. The spectra were recorded in the wavelength range of 200–1100 nm at room temperature. The surface morphology of the ZnO layers was studied by atomic force microscopy (AFM), using the N_TEGRA Prima platform (NT-MDT, Moscow, Russia) and NOVA SPM software license number 1.1.120108. In addition, the layer surfaces and their cross-sections were imaged by scanning electron microscopy (SEM) using SEM Supra 35 (Zeiss, Oberkochen, Germany). The layers were observed at magnifications of 100 × 10^3^, 200 × 10^3^, and 300 × 10^3^ at an accelerating voltage of 2–10 kV. Surface morphology observations and layer thickness measurements were performed in the InLens mode (objective lens). A 10 μm aperture was used for observation. The chemical composition of the layers was investigated using energy dispersive spectroscopy (EDS) UltraDry EDS Detector (Thermo Fisher Scientific EDS, Waltham, MA, USA). Measurements of the chemical composition were carried out with accelerating voltage varying between 3 and 15 kV. The working distance WD for secondary electron SE imaging was 14 mm according to EDS calibration. Apertures of 20 and 30 μm were used for the analysis. The results of the chemical composition were corrected using the Phi-Rho-Z method. The water contact angles of the surfaces of the produced ZnO layers were determined by the goniometric method using the goniometer Ossila (L2004A1, Ossila Ltd., Sheffield, UK). The contact angles were measured in five repetitions for each layer. The obtained values were averaged. To determine the photocatalytic activity of produced ZnO layers, their influence on the decomposition of an organic dye imitating organic impurities in an aqueous solution was examined. Following the Langmuir–Hinshelwood procedure, the tests were carried out on layers corresponding to different durations of the sol aging process. Methylene blue (Merck, Darmstadt-Germany) at a concentration of 5 mg/L was used as a dye. A UV diode M365LP1 operating at a wavelength of *λ* = 365 nm (THORLABS Industriemontagen GmbH & Co. KG, Erfurt, Germany) was used as the light source.

## 3. Results and Discussion

### 3.1. Sol Properties

#### 3.1.1. DLS

**Figure 2:**
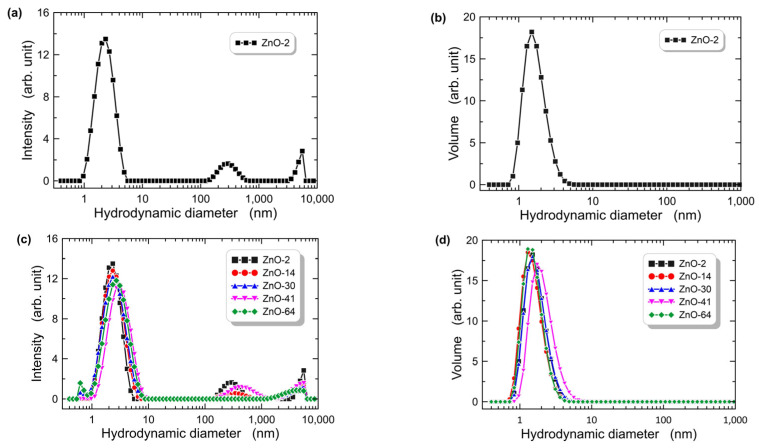
Hydrodynamic diameters of particles in ZnO sol; size distribution by intensity (**a**,**c**), and size distribution by volume (**b**,**d**).

The results of DLS measurements, carried out after 2, 14, 30, 41, and 64 days counted from the day the sol was synthesized, are shown in Figure 2. Samples were marked with labels ZnO-2, ZnO-14, ZnO-30, ZnO-41, and ZnO-64, respectively. Distributions of hydrodynamic diameters of solid particles by the intensity and by volume, respectively, were recorded. The measurement results for the ZnO-2 sol are shown in Figure 2a,b. In both cases, high peaks are visible. Maximum values are reached for the hydrodynamic diameter equal to 2 nm. One can observe in Figure 2a, a low-intensity peak with a maximum for hydrodynamic diameter of 300 nm. The presence of this peak is associated with the formation of agglomerates composed of nanocrystals. The analogous peak is not visible in Figure 2b, which proves the negligible concentration of these agglomerates in the sol.

The results of measurements for longer durations of the sol aging process are shown in Figure 2c,d, respectively. One can see that similar hydrodynamic diameter distributions were recorded, both in terms of intensity and volume. The position of the main peak in the particle size distribution after the intensity is slightly shifted by ca. 1 nm towards larger diameters. In some distributions, one can observe additional peaks above 300 nm, resulting from particle agglomerates, similar to Figure 2a. The particle size distribution by volume is monomodal in each case, which indicates that, despite an increasing duration of aging, the share of agglomerates is negligible. The presented results show that there was a slight increase in particle diameters during the process of sol aging, which indicates slow hydrolysis and condensation processes. The sol was stored at the temperature of 18 °C during the aging. In addition, the resulting particles do not show a clear tendency to form aggregates.

#### 3.1.2. FTIR

**Figure 3:**
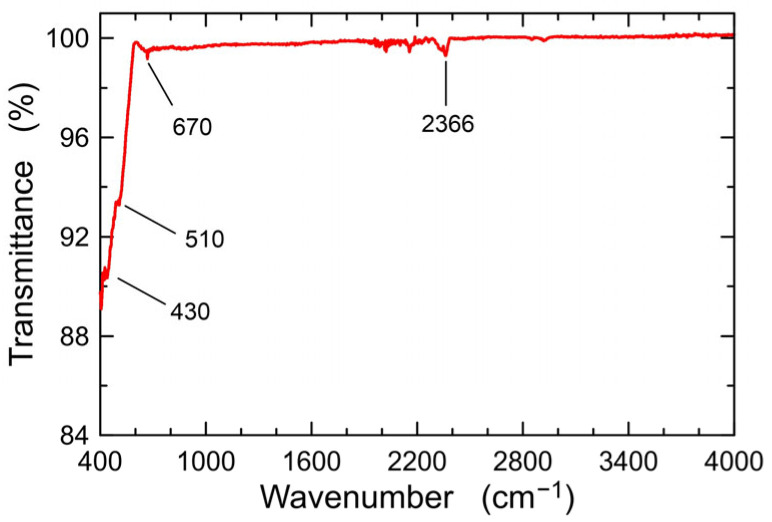
FTIR spectrum for the ZnO powder in the range of 400–4000 cm^−1^.

The FTIR spectrum recorded for the ZnO powder is shown in Figure 3. The interpretation of the visible absorption bands was carried out based on the FTIR spectrum of the same material as presented in Refs [24,40,41,42].

The characteristics show absorption peaks at frequencies of 430, 510, and 670 cm^−1^, which is the effect of Zn-O bond vibrations [42]. Whereas, the absorption band at 2366 cm^−1^ is related to the absorption of atmospheric CO_2_. The spectrum does not reveal the presence of bands resulting from vibrations of bonds in the water molecule, nor the characteristic C=O bands. This means that the applied annealing temperature (485 °C) is suitable for removing organic residues and water from the sol and, consequently, from the powder [40,42].

### 3.2. ZnO Layers

#### 3.2.1. Optical Properties

Thin ZnO layers were produced after the following periods: 2, 14, 30, 41, and 64 days. These periods were measured from the day on which the sol was produced. Glass substrates were coated with sol layers using the dip-coating technique. The substrate withdrawal speed from sol was equal to 4.7 cm/min. Subsequently, the layers were annealed at 485 °C for 1 h. In the following part of the manuscript, these layers will be referred to as ZnO-*t_a_*, where *t_a_* is the aging time. Table 1 summarizes the determined parameters of the produced films.

One can observe from the data in Col. 3, that thickness of ZnO layers varies non-monotonously with the sol aging time. For aging times up to 30 days, an increase in layer thickness is observed, while for longer times, thickness is decreasing. The refractive index (Col. 4) shows exactly the opposite dependence on the aging time of the sol. One can see from the comparison of the data in Col. 3, 4, and 5 that the increase in thickness is accompanied by an increase in porosity and a decrease in refractive index. Subsequent aging results in the opposite direction of changes. Namely, thickness and porosity are decreasing, while the refractive index is increasing.

**Figure 4:**
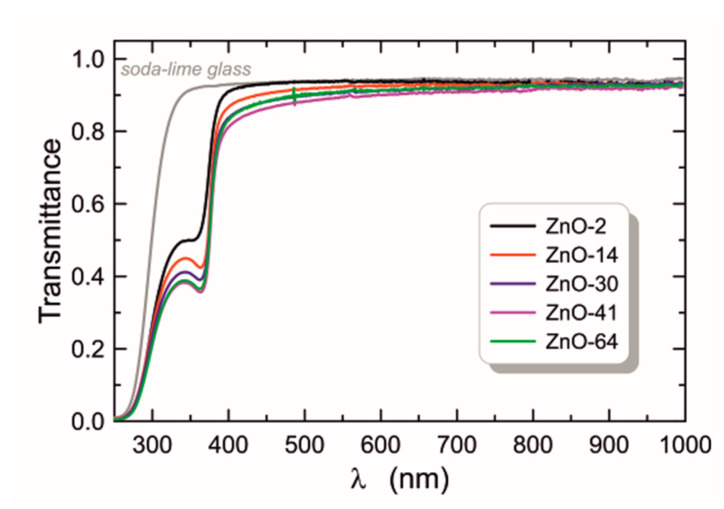
Transmittance characteristics of the layers formed in subsequent days of aging of the ZnO sol.

Transmittance characteristics in the UV-Vis spectral range of all ZnO-*t_a_* structures are presented in Figure 4. The transmittance of a soda-lime glass substrate is also plotted for comparison. In a wide spectral range (*λ* > 400 nm), all structures show slightly lower transmittance than soda-lime glass substrate. A slight decrease in transmittance is the result of the difference between the refractive indices of ZnO and soda-lime glass substrate. The ZnO has a higher refractive index than soda-lime glasses.

Within this spectral range, the transmittance decreases with increasing duration of sol aging time despite the observed decrease in the refractive index (Table 1). The thickness of layers is also initially increasing with the duration of aging and, as will be shown later, their roughness also increases. Thus, as the aging time increases, despite the decrease in refractive index, both thickness and surface roughness have a decisive influence on the reduction in transmission in the spectral range above 400 nm.

One can observe two sharp drops of transmittance in the spectral range below 400 nm. They are the result of strong light absorption in ZnO films. The widths of the optical band gaps were determined using the Tauc relationship [43]:

(1) $$\alpha \cdot h\nu =B{\left(h\nu -E_{g}\right)}^{r},$$

where *α* is the absorption coefficient, *hν* is the photon energy, *B* is the constant, *E_g_* is the determined width of the optical band gap, and *r* = 1/2 results from the direct optical band gap. The absorption coefficient *α* was determined taking into account the light absorption of *A_gs_* in the soda-lime glass substrate, the reflectance of the tested structure *R*, and the fact that, in transmission measurements, the light passes through two layers of the same thickness. In the dip-coating technique, two identical layers are deposited on both sides of the substrate. Hence, the absorption coefficient was calculated using the formula [44]:

(2) $$\alpha =-\frac{1}{2d_{eff}}ln\frac{T}{\left(1-R\right)\left(1-A_{gs}\right)},$$

where *d_eff_* = *d* × (1 − *P*). The porosity *P* was determined from the Lorentz-Lorenz formula [45]:

(3) $$\frac{n^{2}-1}{n^{2}+2}=\left(1-\frac{P}{100\%}\right)\frac{n_{d}^{2}-1}{n_{d}^{2}+2},$$

where *n_d_* = 1.9888 is the refractive index of wurtzite at *λ* = 632.8 nm. Wurtzite is one of the three ZnO crystallographic systems [46]. The wurtzite structure is the only one that is stable at room temperature and atmospheric pressure [47]. The calculated porosities are presented in Table 1 in Col. 5.

**Figure 5:**
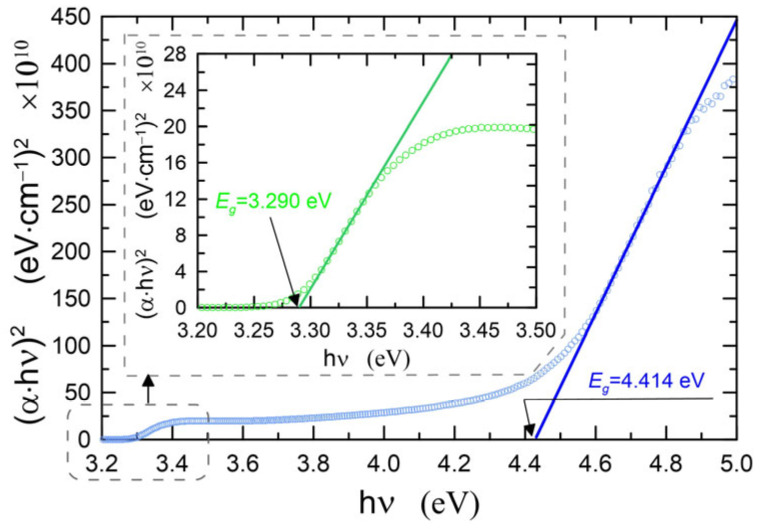
Plots of (*αhν*)^2^ versus photon energy for the structure ZnO-64.

The plot of (*α*·*hν*)^2^ versus photon energy for the ZnO-64 is presented in Figure 5. One can find on this characteristic two ranges where the dependency on photon energy is linear. Zeros of the lines determined by the linear approximation in these ranges are values of optical band gaps. As one can observe, therefore, there are two values of energy. Both of them are different from the value of the optical band gap for the bulk ZnO crystal which equals ca. 3.37 eV. This fact indicates the presence of two types of absorption in our ZnO films. The first one, for photon energy equal to energy band gap, *E_g_^I^* = 4.414 eV > 3.37 eV is connected with the charge transfer excitations in ZnO nanocrystals, and the blue shift of the energy band gap is caused by the quantum size effect. The second absorption type for photon energies below the band gap of bulk ZnO crystal (*E_g_^II^* = 3.290 eV < 3.37 eV) is caused by free exciton. This exciton absorption is responsible for the occurrence of minima in the transmittance characteristics shown in Figure 4 for the wavelength of about 365 nm. Due to the high absorption of photons at this wavelength, a large fraction of the excitations of free excitons may be bound to surface defects. Excitation of free excitons is observed in bulk ZnO, as well as in polycrystalline layers [48,49,50,51].

**Figure 6:**
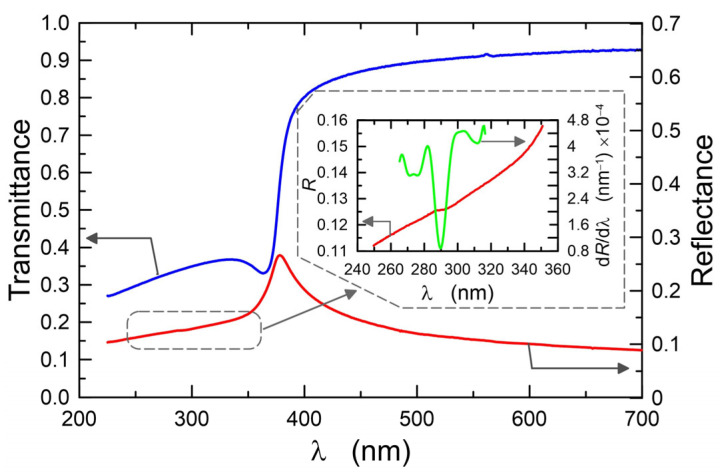
Transmittance and reflectance of ZnO-64 structure.

The determined values of optical band gaps for individual ZnO films are listed in Table 1, Col. 6, and 7, respectively. For each absorption case, the band gaps determined have similar values, and the differences are at the level of 10^−2^ eV. For both absorption bands, the highest *E_g_* value was determined for ZnO-30. In the calculations of the absorption coefficient *α* (Equation (2)), the influence of the soda-lime glass substrate absorption was taken into account. However, since the determined band gap *E_g_^I^* = 4.414 eV lies in the spectral range of the strong absorption of the substrate, we fabricated ZnO films on silica substrates (SiO_2_) to confirm the existence of this absorption band. In Figure 6, the solid blue line shows the transmittance, and the solid red line shows the reflectance. The reflectance characteristics show a clear maximum at the wavelength of *λ* = 377 nm, which corresponds to the excitation wavelength of free excitons (*E_g_^II^* = 3.290 eV). A more detailed analysis also showed slight disturbances in the reflectance characteristics near the wavelength *λ* = 290 nm. An enlarged fragment of the reflectance characteristics is presented in the insert chart. In this spectral range, the reflectance characteristics were differentiated with respect to the wavelength. The d*R*/d*λ* plot has a clear minimum with a value slightly greater than zero at the wavelength of *λ*~290 nm (~4.28 eV). By compensating the slope of the transmittance characteristic, we determined the location of this maximum at the wavelength of *λ* = 282 nm (4.40 eV). The obtained results confirm for the tested ZnO layers the presence of the absorption band at the wavelength of *λ*~281 nm and the optical band gap of *E_g_* = 4.41 eV. In addition, the analysis of the reflectance characteristics showed that its local maxima occur at wavelengths corresponding to the positions of the absorption bands, and that the energies calculated from them exactly correspond to the values of the band gaps determined by the Tauc method.

#### 3.2.2. Quantum Size Effect

The quantity Δ*E* of blue-shift of the energy band gap is in a relationship with a diameter *D* of nanocrystals. For the parabolic energy band near the band gap, the average value of *D* may be estimated from the formula [44,52,53]:

(4) $$\Delta E=\frac{2\pi^{2}\hbar^{2}}{m^{*}D^{2}}-\frac{0.893e^{2}}{\pi \varepsilon \varepsilon_{0}D}-0.248E_{Ry}^{*},$$

where *ħ* is the reduced Planck constant, *ħ* = 1.0545‧10^−34^ J‧s; m* is the reduced effective mass of the electron–hole pair, m* = *m_e_m_h_*/(*m_e_*+*m_h_*); *m_e_* and *m_h_* are the effective mass of the electron and hole, respectively; *ε_0_* is the electric permittivity in a vacuum; *ε* is the dielectric constant for zinc oxide; E^*^_Ry_ is the effective Rydberg energy, E^*^_Ry_ = (m*/(2*ħ*^2^))‧(*e*^2^/4π*ε_0_ε*)^2^. Calculations were carried out by accepting values of *m_e_*, *m_h,_*, and *ε* the same as in Ref. [52]: *m_e_* = 0.24*m_0_*, *m_h_* = 0.45*m_0_*, and *ε* = 3.7 (*m_0_* = 9.11‧10^−31^ kg). Dimensions of crystallites in the ZnO layers were determined by comparing obtained values of *E_g_* with the literature values for the bulk material *E_g_^bulk^* = 3.37 eV [1]. They are presented in Table 1, Col. 9. The diameters of ZnO nanocrystals determined in this way are consistent with the results of DLS measurements.

#### 3.2.3. Urbach Energy

**Figure 7:**
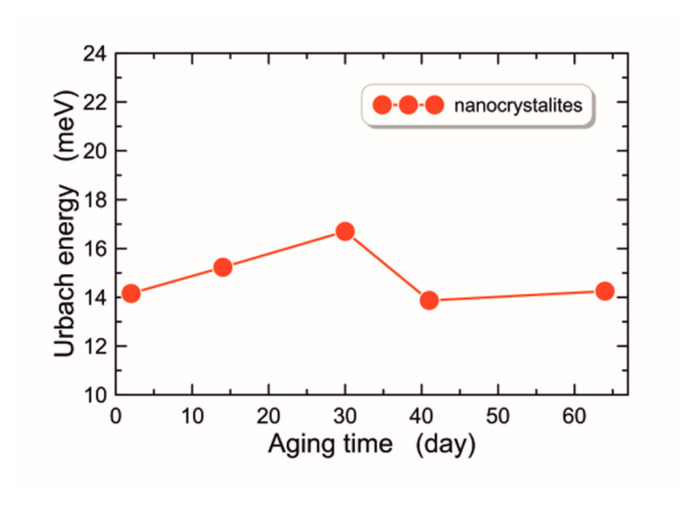
Urbach energy of the films formed in successive days of aging of the ZnO sol.

The data presented in Table 1 (Col. 9) shows that the size of the nanocrystals in ZnO films practically does not change as sol aging time increases. The Urbach energy was determined to track changes in the structure of the layer material. This way, variations in the material structure can be assessed. [54]. The Urbach energy was determined by plotting the relation in the spectral range close to the first absorption band [43]:

(5) $$\alpha \left(h\nu \right)=\alpha_{0}\cdot exp^{\left(\frac{h\nu }{E_{U}}\right)},$$

where *α_0_* is a constant and *E_U_* is the Urbach energy. The high value of the Urbach energy proves that the structure of the material is varying in terms of the size or shape of nanocrystals. The highest Urbach energy values are obtained in amorphous materials or materials with a large number of defects [55,56]. The determined Urbach energy values for individual ZnO films are shown in Figure 7. The presented data show that, since the beginning of the sol aging process during the first 30 days (ZnO-2, ZnO-14 and ZnO-30), the value of the Urbach energy increases. These structures have lower porosity and presumably fewer defects. The Urbach energy for the ZnO-30 structure is *E_U_* = 16.7 meV, and is the highest value among the tested layers. This is probably related to the fact that the ZnO-30 has the highest porosity, and the nanocrystals have the smallest diameters. After this time, there is a clear decrease in the Urbach energy for the ZnO-41 and ZnO-64 layers, which may be related to the lower porosity of the layer material and greater compactness.

#### 3.2.4. SEM and EDS

**Figure 8:**
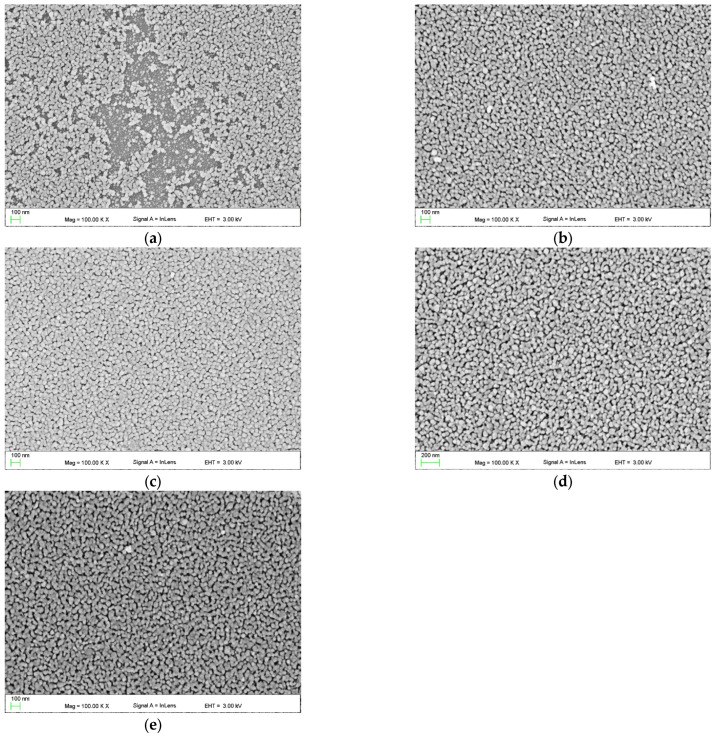
Top view SEM images of the ZnO films, respectively: ZnO-2 (**a**), ZnO-14 (**b**), ZnO-30 (**c**), ZnO-41 (**d**), and ZnO-64 (**e**).

SEM images of the upper surface of ZnO films, recorded in the in-lens mode, are presented in Figure 8. On the other hand, Figure 10 presents SEM images of cross-sections of these layers. Based on these images, we can conclude that zinc oxide layers produced on soda-lime glass substrate have a granular structure. We can also conclude that the ZnO layer is built of grains with a diameter of <70 nm, while from the quantum size effect, it was found that these grains are built of nanocrystals with a diameter less than 0.65 nm. One can observe in Figure 8a that the ZnO-2 layer does not cover uniformly the glass substrate. The SEM image shows the incoherence of the layer and areas of the substrate covered with single ZnO grains.

**Figure 9:**
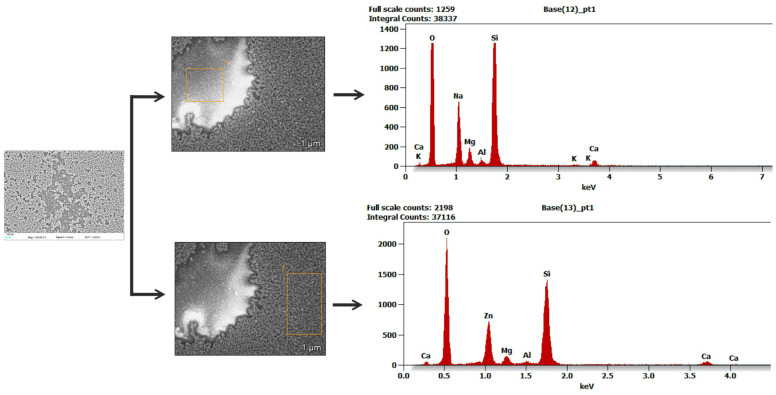
EDS analysis of a thin film of ZnO-2.

A similar inhomogeneity of the substrate coverage is presented in Ref. [57]. Authors reported similar behavior for the layer fabricated after 4 days since the production of their sol. Authors of Ref. [29] also reported such a case for the layer fabricated after 2 days of production of their sol. Obtained EDS spectra are presented in Figure 9. The two peaks at 1.01 keV and 0.53 keV validate the presence of zinc and oxygen, respectively. Besides peaks from the soda-lime glass substrate, the EDS spectra reveal the presence of Zn and O elements, which confirms that the ZnO layer has a grain structure.

**Figure 10:**
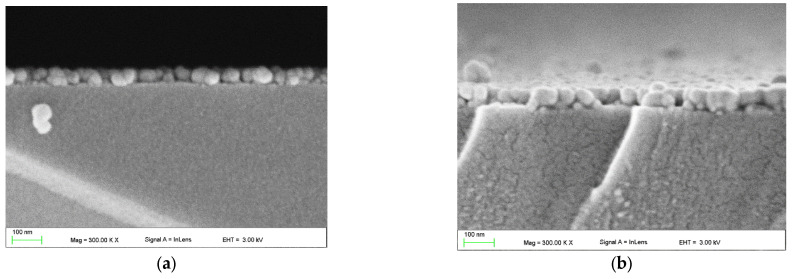

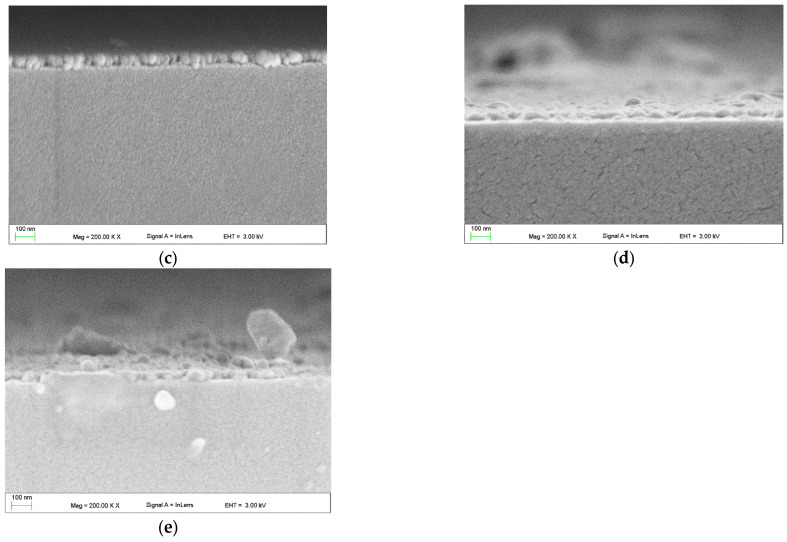
Cross-section SEM images of the ZnO films, respectively: ZnO-2 (**a**), ZnO-14 (**b**), ZnO-30 (**c**), ZnO-41 (**d**), and ZnO-64 (**e**).

One can observe from SEM images (Figure 8b–e) that ZnO layers are uniformly covering the substrates if they are fabricated from the sol which was aged for longer periods. As can be seen, these layers are crack-free and have other structural defects. Moreover, the diameter of the ZnO grains does not change as the duration of the sol aging process increases. Presumably, the sol obtained according to the tested procedure has, immediately after its synthesis, too high surface tension, which in turn lowers the wettability of the substrate. As a result, some areas of the soda-lime glass substrate are not covered with the ZnO layer [58,59]. The uniformity of substrate coverage with the ZnO layer would be improved if the sol was enriched with a surfactant, or some kind of substrate conditioning procedure was applied before the coating process. The images of cross-sections of the ZnO-2, ZnO-14, and ZnO-30 layers (Figure 10a–c) show their grain structure with spherical grain shapes with clearly outlined grain boundaries. In addition, it is noteworthy that the value of film thickness obtained from the analysis of the SEM cross-section image is in good agreement with the one measured using monochromatic ellipsometry. On the other hand, in the SEM images of the ZnO-41 and ZnO-64 layers (Figure 10d,e), grain boundaries are less clear, and the layers are more compact. These observations correlate with the determined decrease in their porosity (Table 1, Col. 5).

#### 3.2.5. AFM

**Figure 11:**
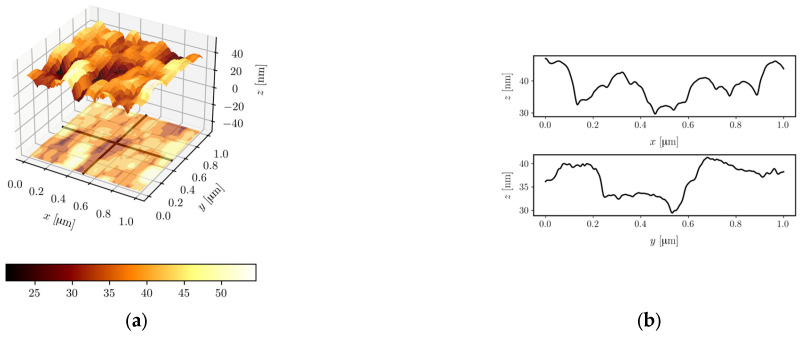

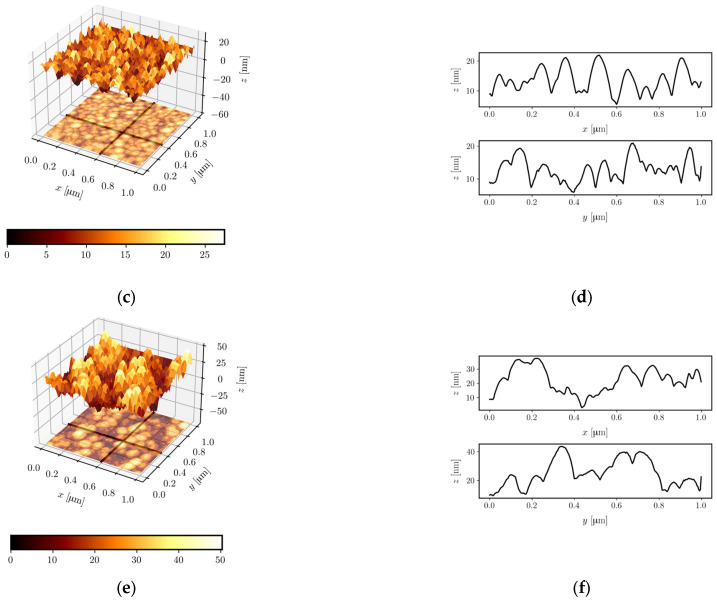
AFM images and corresponding line profiles for ZnO-2 (**a**,**b**), ZnO-30 (**c**,**d**), and ZnO-64 (**e**,**f**) films.

Figure 11 shows the AFM images of the surface of the ZnO-2 (Figure 11a), ZnO-30 (Figure 11c), and ZnO-64 (Figure 11e) layers and their profiles along the selected scans. These images confirm the grainy structure of ZnO films. Moreover, they illustrate the influence of the duration of the sol aging process on the magnitude of the ZnO layer’s surface roughness. The analysis of the ZnO layer’s surface profiles showed that the largest difference in height between the highest and lowest point on the surfaces of 1 × 1 μm^2^ occurs for the ZnO-64 layer and equals 50.3 nm. Calculated values of root mean square (*rms*) surface roughness, over the area of 1 × 1 μm^2^, for the ZnO-2, ZnO-30, and ZnO-64 layers, are 5.02 nm, 3.36 nm, and 8.91 nm, respectively. Similar *rms* values, not so clearly correlated with the sol aging time, were reported in Ref. [23].

Based on results obtained by SEM and AFM methods, we can conclude that the duration of the sol aging process affects the structure of ZnO layers fabricated from that sol. Layers produced from the sol after the shortest duration of the aging process are characterized by discontinuities, while for longer duration, produced layers (ZnO-41, ZnO-64) are characterized by greater compactness. ZnO layers fabricated on every stage of the sol aging process are grainy.

#### 3.2.6. Contact Angles

The measured water contact angles for ZnO layers are presented in Col. 10 of Table 1. One can observe that all layers, regardless of the duration of sol aging, are hydrophilic in nature, as the contact angle in each case is less than 90°. Depending on the time of sol aging, the hydrophilic character of ZnO layers changes slightly, except for the layer made of the longest aged sol. The highest value of the contact angle was obtained for the ZnO-30 layer. It equals 68.53°. Presumably, this is related to the porosity of the layer material, which is also the highest for this layer [60]. The lowest value of the water contact angle, 49.61°, was obtained for the ZnO-64 layer.

#### 3.2.7. Photocatalytic Activity

The photocatalytic activity of the fabricated ZnO layers was investigated by the decomposition of methylene blue (MB) in an aqueous solution. MB was used as a representative impurity. The concentration of the MB solution used was 5 mg/L. ZnO layers, whose photocatalytic activity was investigated, were fabricated on glass substrates of dimensions 20 × 20 × 1 mm^3^. These structures were placed horizontally in a 30 mm diameter beaker filled with 45 mL of MB solution. The solution was agitated magnetically for 30 min in the dark, to achieve equilibrium between the adsorption and desorption processes. Subsequently, the examined structures were irradiated using the LED operating at a central wavelength of *λ* = 365 nm, driven by an electric current of intensity 700 mA. The distance between the ZnO layer and the light source was 6 cm. The total exposure time for each sample was 120 min. The MB degradation process started due to the exposure of submerged ZnO layers to UV light. Subsequently, dye samples of 1 mL were collected from the solution, at regular time intervals of 30 min, and analyzed using UV-Vis spectroscopy. The intensity of the MB absorption band for the collected portion of the dye was measured at the wavelength of *λ* = 664 nm. The intensity of the absorption band for the MB solution recorded at the wavelength of *λ* = 664 nm decreased with the time of its exposure to UV radiation. This is evidence of the degradation of MB dye molecules under the influence of UV irradiation. The processes of dye degradation under the influence of UV radiation are described by the photocatalytic efficiency:

(6) $$\eta \left(\%\right)=\left(1-\frac{c}{c_{0}}\right)\cdot 100\%,$$

and the degradation rate *k*, which, according to the Langmuir–Hinshelwood [61] model, is defined by the equation:

(7) $$ln\left(\frac{c}{c_{0}}\right)=-kt.$$

**Figure 12:**
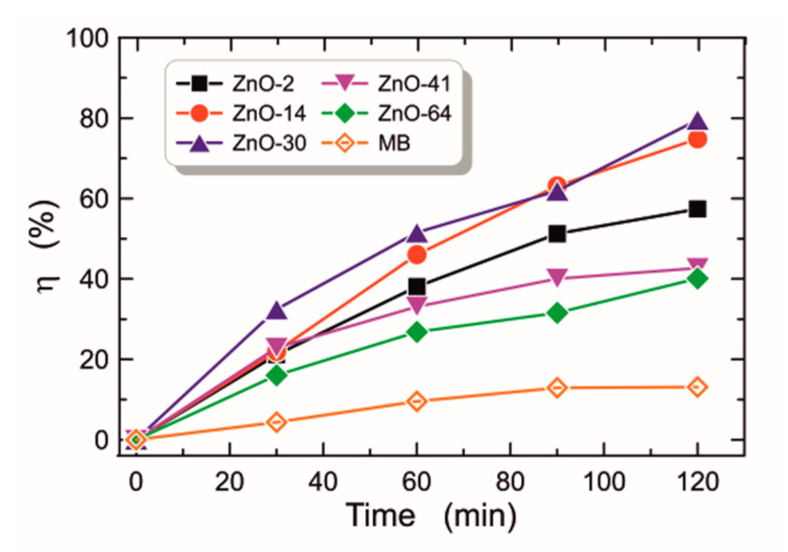
Plots of photodegradation efficiency (*ƞ*) as function time for the degradation of MB dye without (orange empty diamonds) and with the presence of the catalyst.

**Figure 13:**
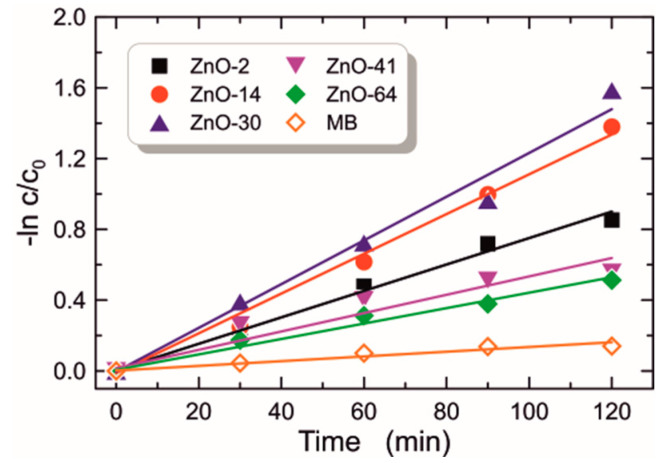
Plots of −ln(*c*/*c_0_*) as a function of reaction time for the degradation of MB dye without (orange empty diamonds) and with the presence of the catalyst.

The degradation rate *k* is also called the first-order reaction rate constant. In the equations above, *c_0_* denotes the concentration of the dye before irradiation, and *c* denotes its concentration after irradiation. Results of the experiment on MB dye degradation are shown in Figure 12 and Figure 13. The characteristic of photocatalytic efficiency as a function of UV irradiation time is presented in Figure 12, while linearized characteristics of the rate of change of the relative MB concentration are presented in Figure 13. As can be seen from the presented data, the presence of the ZnO film during MB dye exposure in each case contributes to the increase in photocatalytic performance. The determined MB dye degradation rates *k* are shown in Col. 11 of Table 1. The MB dye degradation rate without the presence of ZnO is *k* = 1.4 × 10^−3^ min^−1^. According to Ref. [22], differences in photocatalytic activity largely depend on the thickness of the tested layers. However, our research indicates that the highest *k* was obtained for the structure ZnO-30, which has the highest porosity. One can observe that structures ZnO-41 and ZnO-46, which are only slightly thinner than ZnO-30 and thicker than ZnO-2 and ZnO-14, have significantly lower photocatalytic activity than much thinner structures ZnO-2 and ZnO-14. Presumably, the photocatalytic activity depends mainly on the specific surface area (BET) which increases with increasing porosity and diversity of material structure reflected by a value of the Urbach energy. One can observe that the ZnO-30 structure has the highest Urbach energy value (Figure 7), while the structures ZnO-41 and ZnO-46 have lower Urbach energy values, comparable with values for ZnO-41 and ZnO-46. In addition, the ZnO-30 structure has the largest contact angle. These features determine its best photocatalytic properties.

In Table 2 are presented results of the literature survey, which is covering the scope and results of investigations on the influence of the duration of the sol aging process on various physicochemical properties of ZnO thin films. The scope and results presented in this paper are juxtaposed with those obtained by other research groups.

## 4. Conclusions

This paper presents the results of investigations on ZnO layers fabricated using the sol-gel method and dip-coating technique. In the course of sol synthesis, zinc acetate dihydrate was applied as the precursor, while diethanolamine was the stabilizing agent. ZnO layers were produced on soda-lime glass substrates. The layers were annealed at the temperature of 485 °C for 60 min. The maximum duration of sol aging was 64 days. The sol was kept at the temperature of 18 °C throughout the whole aging process. At the end of that process, the gelation process had not ended, and sol was still suitable for coating processes.

Presented studies aimed at the determination of the effect which has the duration of the sol aging process on the properties of produced ZnO layers. They have a thickness varying in the range from 42 nm to 52 nm. The DLS investigations showed that synthesized sol was very stable during the whole aging process. FTIR studies of ZnO powder showed that there are no organic remnants in ZnO layers after the annealing process. The surface morphology of ZnO layers was investigated using SEM and AFM microscopy. Both of these methods revealed that ZnO layers have a granular structure. The spectrophotometric studies showed that the absorption in the UV range has two components: first, below the band gap of bulk ZnO crystal (*E_g_^II^* = 3.290 eV), which is caused by excitation of free exciton and second, connected with the charge transfer excitations in ZnO nanocrystals. The blue energy shift is observed in the second case as a result of the quantum size effect. Moreover, we showed that the optical energy band gaps determined from positions of maxima in reflectance characteristics are equal to those determined using the Tauc method.

The obtained results indicate that there is an optimum duration of the sol-gel aging process that results in the fabrication of layers having extreme values of optical and morphological parameters. All produced ZnO layers showed photocatalytic activity against the aqueous solution of methylene blue dye, however, the most efficient dye degradation was observed in the solution characterized by *k* = 12.3·10^−3^ min^−1^ and *ƞ* = 79.5% for ZnO layers aged for 30 days. It is to be noted that ZnO layers aged for this period have the greatest porosity (*p* = 37.1%), the weakest hydrophilicity (water contact angle 68.53°), and the greatest optical energy band gaps (*E_g_^I^* = 4.485 eV and *E_g_^II^* = 3.300 eV). The high photocatalytic activity of produced ZnO layers allows us to conclude that they may find their applications in environmental applications where the degradation of organic pollutants is required.

## Figures and Tables

**Table 1:** Parameters of the films corresponding to different sol aging times (*d*—thickness of layers; *n*—refractive index of layers; *P*—porosity; *E_g_^II^*—second of the energy band gap; *E_g_^I^*—first of the energy band gap; Δ*E_g_^I^* = *E_g_^I^* − *E_g_^bulk^*; *D*—diameter of nanocrystals; *k*—degradation rate).

Sol Aging Time (Day)	Name	*d* (nm)	*n* (*λ* = 632.8 nm)	*P* (%)	$E_{g}^{\mathrm{II}}$ (eV)	$E_{g}^{I}$ (eV)	Δ*E_g_^I^*	*D* (nm)	Contact Angle (°)	*k* (min^−1^) × 10^−3^
1	2	3	4	5	6	7	8	9	10	11
2	ZnO-2	42	1.631	28.2	3.293	4.455	1.085	0.64	65.27	7.5
14	ZnO-14	45	1.605	30.6	3.290	4.431	1.061	0.65	59.99	11.1
30	ZnO-30	52	1.537	37.1	3.300	4.485	1.115	0.61	68.53	12.3
41	ZnO-41	46.5	1.572	33.7	3.282	4.428	1.058	0.65	63.35	5.4
64	ZnO-64	45.5	1.570	33.9	3.290	4.414	1.044	0.68	49.61	4.4

**Table 2:** Comparison of the present article with published articles based on sol aging time and thin layers ZnO.

Sol Aging Time	Sol Composition	Methods, T (°C), Substrates	Results	Ref.
0–36 h	zinc acetate dihydrate, monoethanolamine, 2-methoxyethanol	spin-coating, 300, 500, glass substrates	the optimal aging time is 24 h, the ZnO films were of high quality and had the best properties (transmission in the UV-Vis range, bandgap broadening)	[30]
0–13 days	zinc acetate dihydrate, methanol	dip-coating (multilayers), 500, glass substrates	the optimal aging time is 7 days, the layers were characterized by high transmittance (above 70% in the visible range) and a smooth surface	[27]
0–30 days	zinc acetate dihydrate, diethanolamine, ethanol	dip-coating, 450, glass substrates	the longer the sol aging time, the ZnO layers having better transmittance in the UV-Vis range (up to 96%), increased optical band gap Eg, and improved smoothness	[23]
2–64 days	zinc acetate dihydrate, diethanolamine, ethanol	dip-coating (monolayer), 485, glass substrates	the optimal sol aging time is 30 days, the ZnO layers are characterized by the best optical and photocatalytic properties, after this time the layers have weaker optical and photocatalytic properties	this work

## Data Availability

Data sharing is not applicable to this article.
